# Revisiting the Local Structure in Ge-Sb-Te based Chalcogenide Superlattices

**DOI:** 10.1038/srep22353

**Published:** 2016-03-01

**Authors:** Barbara Casarin, Antonio Caretta, Jamo Momand, Bart J. Kooi, Marcel A. Verheijen, Valeria Bragaglia, Raffaella Calarco, Marina Chukalina, Xiaoming Yu, John Robertson, Felix R. L. Lange, Matthias Wuttig, Andrea Redaelli, Enrico Varesi, Fulvio Parmigiani, Marco Malvestuto

**Affiliations:** 1 Università degli Studi di Trieste, Via A. Valerio 2, 34127, Trieste, Italy; 2 Elettra-Sincrotrone Trieste S.C.p.A. Strada Statale 14-km 163.5 in AREA Science Park 34149, Basovizza, Trieste, Italy; 3 Zernike Institute for Advanced Materials, University of Groningen, Groningen 9747, AG, The Netherlands; 4 Department of Applied Physics, Eindhoven University of Technology, P. O. Box 513 5600, MB Eindhoven, The Netherlands; 5 Paul-Drude-Institut für Festkörperelektronik, Hausvogteiplatz 5-7 10117, Berlin, Germany; 6 Russian Academy of Sciences, Institute of Microelectronics Technology and High Purity Materials, Moscow, Russia; 7 Engineering Department, Cambridge University, Cambridge CB2 1PZ, UK; 8 Institute of Physics, RWTH Aachen University, 52056, Aachen, Germany; 9 Micron Semiconductor Italia S.r.l., Via C. Olivetti, 2, 20864, Agrate Brianza, MB, Italy; 10 International Faculty, University of Cologne, 50937 Cologne, Germany

## Abstract

The technological success of phase-change materials in the field of data storage and functional systems stems from their distinctive electronic and structural peculiarities on the nanoscale. Recently, superlattice structures have been demonstrated to dramatically improve the optical and electrical performances of these chalcogenide based phase-change materials. In this perspective, unravelling the atomistic structure that originates the improvements in switching time and switching energy is paramount in order to design nanoscale structures with even enhanced functional properties. This study reveals a high- resolution atomistic insight of the [GeTe/Sb_2_Te_3_] interfacial structure by means of Extended X-Ray Absorption Fine Structure spectroscopy and Transmission Electron Microscopy. Based on our results we propose a consistent novel structure for this kind of chalcogenide superlattices.

The need for fast and efficient management of information stimulates research on materials that can be switched on nanometer length scales and sub-nanosecond time scales. Phase-Change materials (PCMs) possess a unique property portfolio, which is ideally suited for memory device applications^1^,^2^,^3^,^4^,^5^,^6^. A PCM is identified by its ability of switching rapidly and reversibly between a crystalline and an amorphous state, where the amorphous state is obtained by melting the crystalline state followed by rapid quenching. These two states significantly differ in their properties, such as the optical reflectivity as well as the electrical conductivity. The phase transformation is in general triggered by thermal heating, or by either electrical and optical pulses of different time duration and amplitude. The large contrast in reflectivity between these two states lays at the base of already working PCM-based optical rewritable media devices-like DVDs or Blu-Ray Disc-where information is encoded as amorphous marks in a crystalline background. The contrast in resistivity could be exploited in the next generation of electronic solid-state memories based on PCMs, which might replace the current leading storage technologies, namely FLASH and magnetic disks. Furthermore, these materials could be employed in displays or data visualization applications by combining both their optical and electronic property modulations^7^. Hence, a lot of interest and effort is currently devoted to uncover the complex physical origin of the high contrast between the two phases^8^,^9^,^10^, as well as of the atomistic representation of the switching mechanism^11^,^12^,^13^.

Most PCMs are compounds located in the ternary Ge:Sb:Te-phase diagram^6^. In particular, alloys formed by the mixture of GeTe^14^,^15^ and Sb_2_Te_3_^16^ show high distinguishable phases, stability, cyclability and scalability^17^. Nanostructures positively impact the physical properties of PCMs with respect to the single crystal analogues. Within this framework, “superlattice-like” chalcogenides have been developed, where nano-scale GeTe and Sb_2_Te_3_ units are alternatively deposited at room temperature^18^,^19^,^20^,^21^. They are shown to switch between a polycrystalline and an amorphous phase and to operate with lower power threshold and faster switching time. Indeed, such stacking layout might cause a reduction of thermal transport, which enables a higher temperature raise for the same absorbed power^18^,^19^,^20^,^21^. Recently, by increasing the deposition temperature (~250 °C) and by reducing the GeTe sublayer thickness (down to 1 nm), interfacial PCMs (iPCMs) have been designed^22^. The improvements obtained for this particular structure are explained by a refined atomic switching process model, circumventing pure mass melting^23^,^24^,^25^,^26^,^27^. The underlying idea is that in 1 nm only two GeTe bilayers (BLs) are present and are the active ones. As a consequence, a change in the local Ge atomic bonding occurs and confines the motion in one dimension. The two reversible phases of iPCMs are thus claimed to be both crystalline. Correspondingly a reduction of the entropy variation with respect to a classic order-disorder transition is expected. Hence, since the GeTe sublayer plays a crucial role in defining the fundamental phase-change microscopic properties and since a nominal 1 nm of GeTe corresponds closely to 3 BLs^28^, establishing the GeTe atomistic nature as well as its interface with Sb_2_Te_3_ is a mandatory and pivotal point.

The present structural study falls in this context by assessing the in-depth atomistic details of the as-grown highly-textured epitaxial [GeTe (1 nm)/Sb_2_Te_3_ (3 nm)] superlattice-herein named chalcogenide superlattice (CSL)-which is grown fully crystalline at high temperature. Most of structural reports are based upon Transmission Electron Microscopy (TEM) and (non-resonant) X-ray diffraction studies. X-ray diffraction probes the two-body correlation function over tens of nanometers. Yet, the atomic arrangement can only be derived from a comparison of model simulations with the measured data, which presents significant challenges for an unambiguous identification of the atomic arrangement. Conversely, TEM is capable of providing high-resolution structural information but is characterized by a limited atomic contrast and resolution (up to 1 Å). These shortcomings create a need for complementary techniques such as EXAFS. Due to the correlation between the absorber and the backscatter through the emitted photoelectrons excited by X-rays, EXAFS probes the local structure around the selected photo-absorber, in terms of number of neighbouring atoms N, average bond length between absorbing-neighbouring atoms 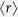 and variance 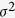. Since EXAFS is an element-specific technique, in these systems we may independently achieve information on the individual first nearest neighbours and next nearest neighbours atomic pairs.

In next sections we report on the Ge and Sb local atomic environment at the interface between the GeTe and Sb_2_Te_3_ layers. Supported by high-resolution TEM and an extended Morlet Wavelet-Transform analysis, EXAFS reveals that the GeTe BLs are not isolated but intercalated within 1 Sb_2_Te_3_ quintuple layer (QL), where the external Sb layers are intermixed with Ge atoms.

## Materials and Experimental

A CSL sample of [GeTe (1 nm)/Sb_2_Te_3_ (3 nm)]_15_ with total thickness 60 nm was deposited on Sb-passivated Si(111) surfaces^29^, at a substrate temperature of 230 °C, by means of molecular beam epitaxy (MBE). The film was finally capped to avoid oxidation. The thermodynamically controlled growth has been shown to lead to high-quality superlattices, as in our case^28^,^30^. GeTe^31^ and Sb_2_Te_3_^32^ single crystals were used as reference samples. Further details about the growth processes for all the samples can be found in the Supplementary Information. The experimental work was carried out on the SAMBA beamline at SOLEIL Synchrotron and on the B18 beamline at Diamond Light Source^33^,^34^. The EXAFS spectra were collected in fluorescence yield mode at room temperature.

## Results and Discussion

**Figure 1:**
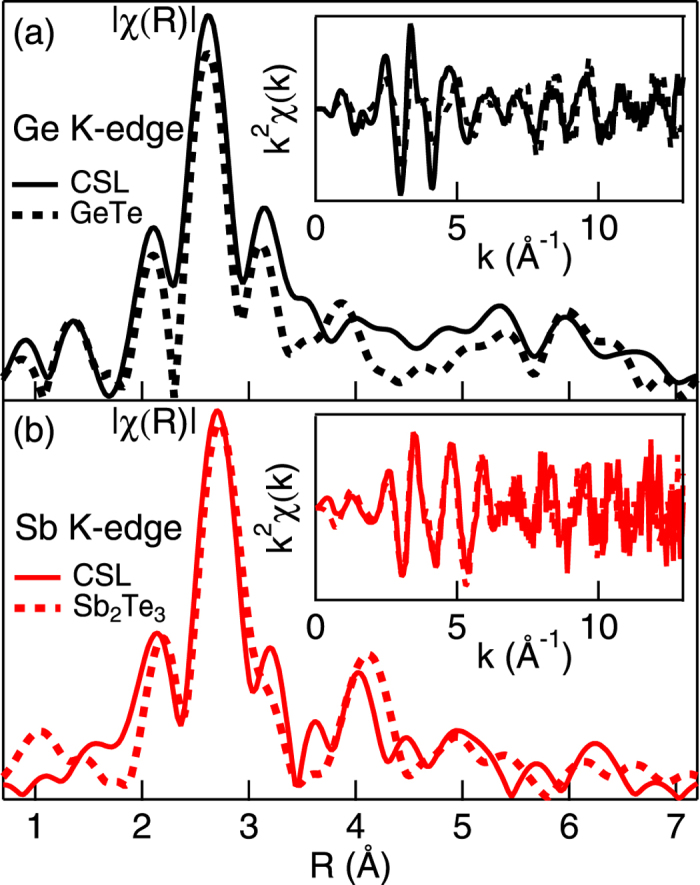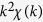 EXAFS signals (inset) and corresponding Fourier Transform magnitudes 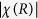 (main panel) of the CSL sample (continuos curves) and of the reference crystal samples (dashed lines)-GeTe and Sb_2_Te_3_, respectively-at: (**a**) Ge *K*-edge; (**b**) Sb *K*-edge.

Although the nearby environment of Ge and Sb atoms in the CSL resembles that of bulk GeTe and Sb_2_Te_3_, appreciable distortions are observed in particular beyond the first shell. Inset in Fig. 1(a) shows the Ge K-edge EXAFS data in the photon momentum k range (0–13.5 Å^−1^), collected for the bulk GeTe and the CSL samples. The comparison reveals a different k-dependent damping and a slight phase shifting of the CSL oscillations with respect to the bulk signal. Such differences can be better appreciated by looking at the EXAFS signals Fourier Transforms (FTs) 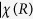
| in R-space (Fig. 1(a) main panel). These profiles yield a raw representation of the local structure, where the photo-absorber is centred at R = 0 Å. Both the 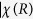
| spectra display a prominent double peaked feature at R = 2.7−3.2 Å, stemming from the two photoelectron scattering paths with the first nearest neighbours. In analogy to GeTe single crystal, these paths link Ge to Te atoms. The weaker peaks and shoulders at higher distances can be attributed to the contribution of the next nearest neighbours and multiple scattering effects. In particular, a noticeable difference of the signals in the 3.2−5 Å radial region reveals a different environment of Ge atoms in the CSL GeTe layers with respect to the stoichiometric GeTe structure. Figure 1(b) inset displays the EXAFS data at the Sb K-edge. A damping-free overlap of the oscillations is visible, leading to a clear proximity in the relative 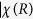
| profiles of the double peaked structure at R = 2.9−3.2 Å. Nevertheless, also in this case, the correspondence of the two EXAFS spectra decreases when moving towards higher R. As far as the nearest neighbour spacing is concerned, the similarity of CSL with the corresponding single crystals used for reference (GeTe and Sb_2_Te_3_) is preserved. Having established a qualitative analysis of the Sb and Ge K-edge 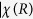
| profiles, it is justified to assume that the average local atomic details of the Ge and Sb photo absorbers in the CSL layers is not exactly compatible with bulk crystalline structure of GeTe and Sb_2_Te_3_ beyond the nearest neighbour range. The observed differences might help to provide important insights into the atomic arrangement of Ge and Sb atoms in the CSL structure.

**Figure 2:**
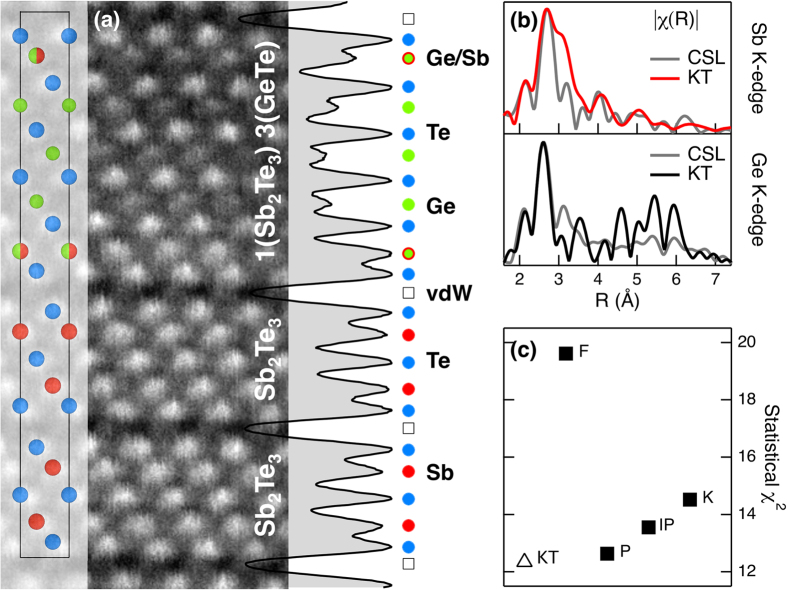
(**a**) High resolution TEM image of the CSL sample, with overlayed experimental-based averaged structure model. Sb, Te and Ge atoms are denoted with red, blue and green circles, respectively. Half-colored circles indicate an ideal 50% intermixing of Sb and Ge atoms, as suggested by a quantitative analysis of the present image (black profile). (**b**) 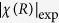 of the experimental EXAFS data at Ge and Sb K-edge (grey curves) in comparison with the ab-initio simulated 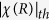 (Ge-black, Sb-red curves) of the Kooi TEM (KT) model shown in (**a**). (**c**) 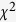-values from a comparison between theoretical models^24^ and experimental CSL data.

In search for a valid structural model of the [GeTe/Sb_2_Te_3_] interface, we considered the representative crystalline structure revealed by a high-angle annular dark-field imaging scanning TEM study of our CSL sample^28^. Figure 2(a) displays the resulting close-up atomic resolution image with an overlying schematic of the unit cell. This model, herein named Kooi TEM (KT), matches the CSL layered structure reasonably well. Interestingly, the related quantitative investigation on the high-resolution image-black profile^28^-clearly shows two main tendencies: (i) the structure assumes that *m* GeTe units are intercalated within one single Sb_2_Te_3_ QL [Te-Sb-Te-Sb-Te], and that the whole block can be coupled via van der Waals (vdW) gaps to (*n-1*) Sb_2_Te_3_ QLs, like in natural GST alloys^35^. In particular, here *m *= 3 and *n *= 2; (ii) the external Sb layers in the block are intermixed with Ge atoms, presumably due to inter-diffusion.

Based on this structural model for the [GeTe/Sb_2_Te_3_] stack, 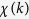 signals at the Ge and Sb K-edge were computed with an ab-initio approach by means of the feff6 code^36^. The simulated spectra were calculated considering all non-equivalent Ge and Sb atomic positions for the absorber and then including single and multiple backscattering paths for a cluster of 8 Å. The pseudo-fitting parameters were set to 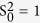, 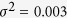, ΔR_eff_ = 0, ΔE_0_ = 0. Figure 2 (b) shows the FT[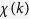] moduli on the R-space at Ge (black curves) and Sb (red curves) K-edge compared to the CSL experimental 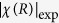 spectra (grey curves). The calculated 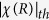 spectra, normalized to the main peak, significantly mirror the main features of the experimental 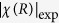 profiles, up to ~6−7 Å for both the Ge and Sb K-edge. For consistency, we computed the simulation also considering four other stacking sequences proposed in literature for similar systems, namely Ferro Ge-Te (F), Petrov (P), Inverted Petrov (IP) and Kooi (K)^24^. The models unit cells and the 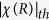 profiles can be found in Fig. 1S(a) of the Supplementary Information. Although these simulations are affected by the unavoidable approximation of the ab-initio calculation, a good agreement between all the models and the experimental data is appreciable as far as the first nearest neigbor range is taken into account. On the other hand, it is to be noted that the second and third neighbor structure plays a crucial role in distinguishing the models. Thus, a 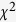 comparison was implemented with an IGOR pro built-in function^37^, yielding an estimate of how representative a single model is with respect to the experimentally observed structure. The 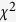 values relative to the two K-edges were summed up for each model-experiment pair in order to obtain a correlation. The results of this analysis are reported in Fig. 2(c). The KT model has the lowest 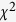 value and, interestingly, the 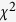 value provided by the Petrov (P) model is also close to the minimum. This is probably due to the fact that this structure can be viewed as a KT stacking sequence with an extra Ge layer between the two central Te layers (Fig. 1S(b)). Consequently, the comparison discussed above suggests that the KT structure, being experimental-based and being noticeably more consistent at high R-values with respect to the other models, is a good starting point for a refinement of the quantitative EXAFS fitting analysis.

**Figure 3:**
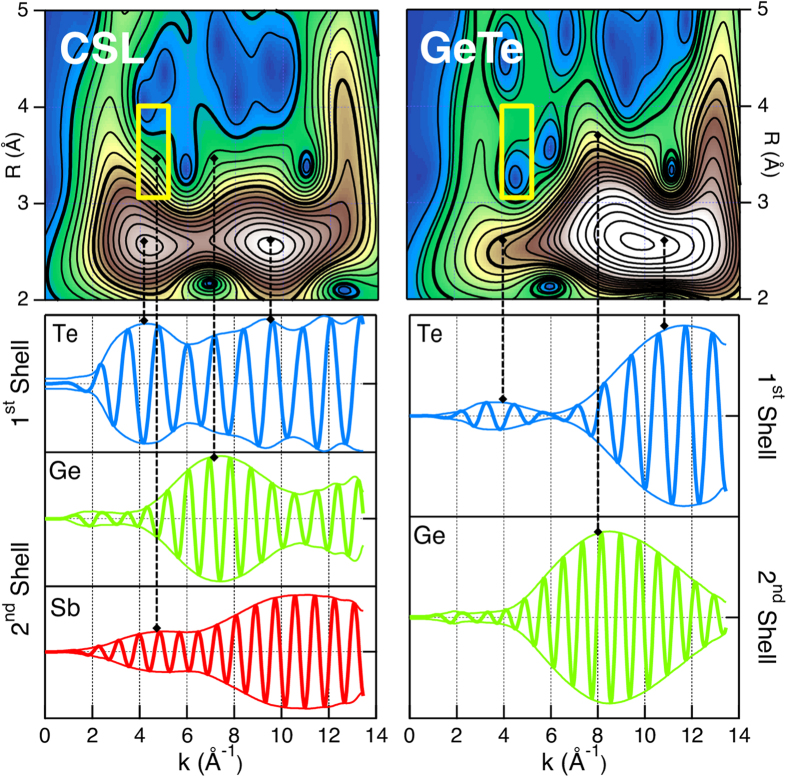
Upper panels: 2D plot of the Morlet Wavelet-Transform analysis. Lower panels: simulation of the backscattering paths corresponding to the 1^*st*^ and 2^*nd*^ shells, for the KT and the GeTe single crystal^39^ models. Vertical dashed lines are guides to the eye. The maxima of the envelope of the model backscattering signals contribute to specific regions (yellow boxes) of the experimental WT. The comparison with the model signals helps to decompose the experimental WT.

Furthermore, in order to corroborate the qualitative observations of the raw data and of the *χ*^2^ comparison, we implemented a continuous wavelet transform (WT) analysis^38^ on the CSL and GeTe samples at the Ge K-edge. The WT analysis is able to resolve the k-dependence of the absorption signal, having a simultaneous resolution in R-space. In this way, the contribution from atoms of different elements at the same distance from the absorber can be in principle distinguished. The upper panels in Fig. 3 show the resulting 2D plot of the WT analysis, using k^3^-weighted spectra and a Morlet function as kernel. It is to be noted that, at a first glance, the main difference between single crystal reference and superlattice signals output is visible in the radial region corresponding to the first shell, namely 2−3 Å. Two contributions at 4 Å^−1^ and 10 Å^−1^ are clearly resolved in the CSL, while in GeTe the first ridge is less intense. Moving towards the second shell radial region 3−4 Å, a shoulder appears between 4−6 Å in CSL, replacing the intensity-hole present in GeTe. In order to understand these qualitative differences, we performed simulations of the backscattering paths 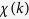 signals corresponding to the first and second shells, according to the method above mentioned. For CSL and for GeTe we used the KT model and the structure described by Nonaka *et al.*^39^, respectively. Figure 3 lower panels display these simulations. The dashed vertical lines associate the peaks of the various k^3^-weighted 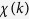 envelopes to the region with corresponding intensity in the 2D WT map. Regarding the first shell, the envelope amplitudes match with experimental data. The difference between CSL and GeTe, can be ascribed to the inclusion of multiple feff calculations referred to non-equivalent Ge sites for CSL, rather than of the single one for GeTe case. Therefore, more than one type of Te local environment with respect to Ge atoms might be present. Focussing on the second shell, in particular in the k-region where appreciable dissimilarities with the GeTe case are visible, the contributions of Ge-Ge backscattering paths agree in both images. Interestingly, the intensity hole observed in the momentum-distance yellow box (4−5.5 Å^−1^) × (3−4 Å) of the single crystal case is completely filled for the CSL case. Important to note is that, by looking at the simulated profile of Sb neighbouring atoms, a peak of the envelope is visible at low k values. Therefore, this observation upon a simple change on the visualization of the raw data can help in identifying the presence of a backscatter element different from Ge and Te in the next nearest neighbours environment of Ge absorbing atoms, (see also Supplementary Information).

**Figure 4:**
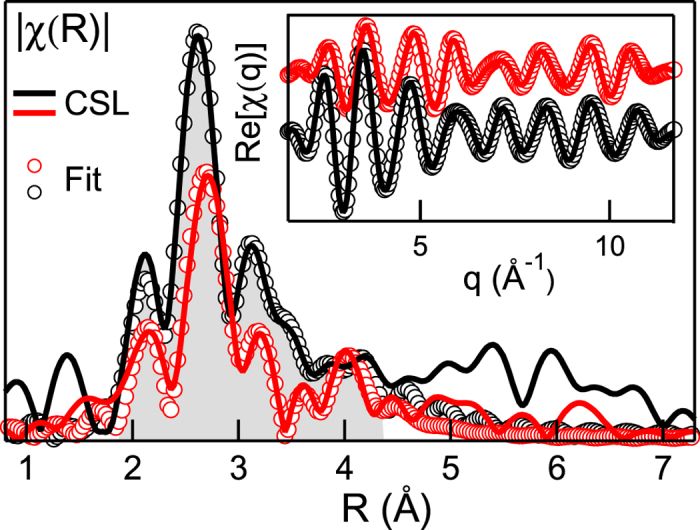
FTs moduli and (inset) back FT real parts of experimental EXAFS profiles at Ge and Sb *K*-edge of CSL and best fit results. Shadowed-grey region corresponds to the R-hanning window (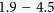 Å) considered for the back FT extraction and fitting.

**Figure 5:**
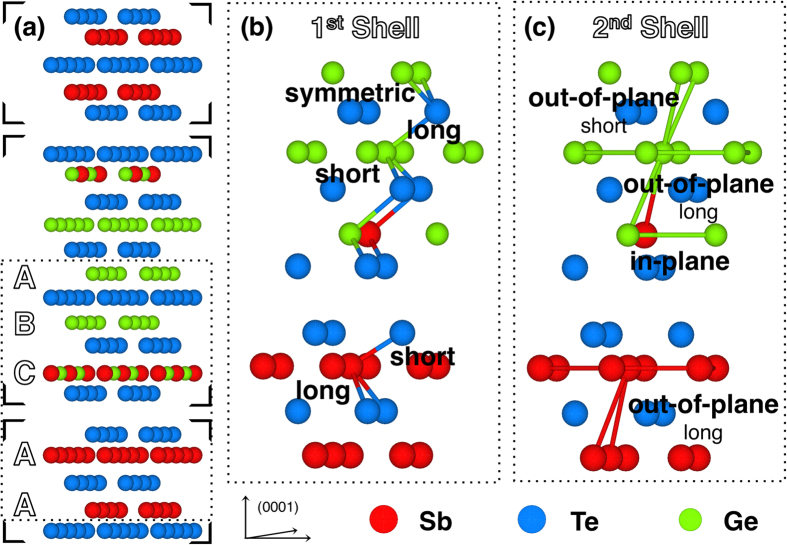
(**a**) Prospective view of the average structure model KT used in the fitting procedure. It presents an ideal 50% intermixing with Ge atoms in the Sb layer in front of the QLs. (**b**,**c**) Close-up of the dashed zone in panel (**a**) with highlighted distances relative to the 1^*st*^ and 2^*nd*^ shells, respectively. In particular, the marked Sb and Ge atoms represent the different absorber inequivalent positions A, B and C.

The experimental-based KT model was used to fit the back FT of the CSL sample EXAFS 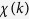 oscillations (Fig. 4 inset). In the single building-block, made up of 3 GeTe BLs intercalated within 1 Sb_2_Te_3_ QL, the Sb-layers locate preferentially close to the edges^35^. Here, these Sb-layers are considered with an ideal 50% intermixing of Ge atoms, schematized in Fig. 2(a) with half-colored balls. Henceforth the following nomenclature will be used for recalling the atomic positions in the unit cell as depicted in Fig. 5: (i) A site, for Ge or Sb atoms in the center of their respective building-block (GeTe or QL); (ii) B site, for Ge atoms close to the intermixed layer in the main building-block; (iii) C site, for Ge or Sb atoms in the intermixed layer. In order to improve the fitting reliability and to decrease the number of variables, we performed a co-refinement procedure of the CSL experimental data at Ge and Sb K-edge, using the Artemis code^36^. According to the atomic element populations in the unit cell, the feff calculations relative to A, B and C atomic sites were included in the procedure, with different weights (see Supplementary Information). Figure 4 shows the Ge and Sb K-edge spectra and their best fits in R (main panel) and q (inset) space. The R_*factor*_ represents a measure of a fit effectiveness, being the sum of the square of the residuals between measured and model data, normalized to the magnitude of the experimental data. Furthermore, distances with a separation higher than *δ*R = *π*/2*k*_*max*_ can be resolved by EXAFS spectroscopy^40^. In the CSL case of our investigation, R_*factor*_ = 0.0087 and the experiment resolution is given by *δ*R = 0.14 Å.

Table 1 lists the interatomic distances of CSL, as a result of the co-refinement fitting procedure with the KT structure model. We identify two concentrically coordinated spheres surrounding the Ge and Sb absorbers with different radii. The first shell includes the short and long out-of-plane bonds between the absorbers and the Te atoms, which are schematized in Fig. 5(b). A quick look at the interatomic distances in Table 1 indicates the presence of structural distortions with respect to the bulk parent compounds. While Sb sites, regardless of the Sb atomic position, tend to keep a robust environment similar to that of bulk Sb_2_Te_3_, Ge sites show increasing distortion depending on the distance from the interfacial layer. We will focus now on the first shell distances. As shown in Table 1, all the Sb-Te distances have a strong similarity with bulk Sb_2_Te_3_ (see Table 2S in Supplementary Information for reference), within the experimental error uncertainty. On the contrary, for the Ge-Te distances we observe three non-equivalent Ge sites. In particular, A-Ge presents a 6-fold symmetry that is lost in the other two cases; B-Ge has a bulk GeTe correspondence (see Table 3S in Supplementary Information for reference). Interesting to note is that C-Ge resembles instead the Sb-Te distances in the QLs. The second shell is composed by the in-plane and out-of-plane bonds of the absorbers with Ge and Sb atoms, which are schematized in Fig. 5(c). X–X defines the pairs Ge-Ge, Ge-Sb and Sb-Sb. The in-plane distance (X–X_*in−p*_) coincides with the lattice parameter of the CSL unit cell for all layers. The resulting value is smaller than the single crystal references ones. Essentially, except for the central A-Ge, the second shell distances are consistent with bulk Sb_2_Te_3_. The Ge-Te and Ge-Ge planes in the CSL internal block are slightly vertically closer than in bulk GeTe. The intermixed Sb(Ge) layers preserve the distances as in QLs. The distortions observed on the CSL structure suggest that the physical properties, and in particular the bonding character, is substantially different from what predicted by idealized CSL. The observation of three different types of Ge sites implies that also their chemical bonding is different. The undistorted 6-fold Ge-Te bonding is in strict contrast with the GeTe bulk case where Peierls distortions form distinct shorter and longer bonds. This observation opens up a scenario where the undistorted GeTe site in the CSL structure determines a pronounced electron delocalisation and 0 energy gap at E_*F*_ which characterises resonant bonding^41^,^42^. Such an atomic structural evidence could also rationalise the observed metallic state of our CSL samples. The distorted B and C Ge sites may lead to a significantly increased electronic static polarizability and in turn to a ferroelectric response.

## Conclusions

Finally, a representative high-quality [GeTe (1 nm)/Sb_2_Te_3_ (3 nm)]_15_ superlattice sample is probed by means of EXAFS spectroscopy to unveil the as-grown crystalline state structure at the interface of the two materials. The observed distortion of the lattice, the vdW gap shift and the Ge(Sb) intermixing should be considered as worthwhile features of these as-grown CSLs, since they would possibly affect the phase-change performances, in terms of electrical switching and activation energy. In this regard, our results-combining an optimized growth process and a careful atomistic structural characterization-prospect for CSLs a phase-change mechanism more complex than a oversimplified atomic Ge flipping.

## Additional Information

**How to cite this article**: Casarin, B. *et al.* Revisiting the Local Structure in Ge-Sb-Te based Chalcogenide Superlattices. *Sci. Rep.* **6**, 22353; doi: 10.1038/srep22353 (2016).

## Supplementary Material

Supplementary Information

## Figures and Tables

**Table 1:**
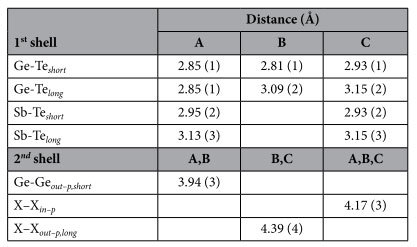


Interatomic distances relative to 1^*st*^ and 2^*nd*^ shell of CSL, as a result of the co-refinement fitting procedure. The atomic positions are identified with A, B and C, as depicted in Fig. 5. X–X defines the pairs: Ge-Ge, Ge-Sb and Sb-Sb.

## References

[b1] Wuttig M. & Yamada N. Phase-Change Materials for rewriteable data storage. Nat. Mater. 6, 824–832 (2007). 17972937 10.1038/nmat2009

[b2] Wuttig M. Phase-change materials: Towards a universal memory? Nat. Mater. 4, 265–266 (2005). 15875300 10.1038/nmat1359

[b3] Lankhorst M. H. R., Ketelaars B. W. S. M. M. & Wolters R. A. M. Low-cost and nanoscale non-volatile memory concept for future silicon chips. Nat. Mater. 4, 347–352 (2005). 15765107 10.1038/nmat1350

[b4] Caldwell M. A., Jeyasingh R. G. D., Wong H.-S. P. & Milliron D. J. Nanoscale phase change memory materials. Nanoscale 4, 4382–4392 (2012). 22740071 10.1039/c2nr30541k

[b5] Ovshinsky S. R. Reversible electrical switching phenomena in disordered structures. Phys. Rev. Lett. 21, 1450–1453 (1968).

[b6] Deringer V. L., Dronskowski R. & Wuttig M. Microscopic complexity in phase-change materials and its role for applications. Adv. Funct. Mater. 25, 6343–6359 (2015).

[b7] Hosseini P., Wright C. D. & Bhaskaran H. An optoelectronic framework enabled by low-dimensional phase-change films. Nature 511, 206–211 (2014). 25008527 10.1038/nature13487

[b8] Akola J. & Jones R. O. Structural phase transitions on the nanoscale: The crucial pattern in the phase-change materials Ge_2_Sb_2_Te_5_ and GeTe. Phys. Rev. B 76, 235201 (2007).

[b9] Huang B. & Robertson J. Bonding origin of optical contrast in phase-change memory materials. Phys. Rev. B 81, 081204 (2010).

[b10] Siegrist T. *et al.* Disorder-induced localization in crystalline phase-change materials. Nat. Mater. 10, 202–208 (2011). 21217692 10.1038/nmat2934

[b11] Lee B.-S. *et al.* Observation of the role of subcritical nuclei in crystallization of a glassy solid. Science 326, 980–984 (2009). 19965508 10.1126/science.1177483

[b12] Kolobov A. V., Krbal M., Fons P., Tominaga J. & Uruga T. Distortion-triggered loss of long-range order in solids with bonding energy hierarchy. Nat. Chem. 3, 311–316 (2011). 21430691 10.1038/nchem.1007

[b13] Zhang W. *et al.* Role of vacancies in metal-insulator transitions of crystalline phase-change materials. Nat. Mater. 11, 952–956 (2012). 23064498 10.1038/nmat3456

[b14] Liebmann M. *et al.* Giant Rashba-Type Spin Splitting in Ferroelectric GeTe(111). Adv. Mater. (2015). 10.1002/adma.201503459 26599640

[b15] Klemm W. & Frischmuth G. Das system Germanium–Tellur. Z. anorg. allg. Chem. 218, 249–251 (1934).

[b16] Zhang H. *et al.* Topological insulators in Bi_2_Se_3_, Bi_2_Te_3_ and Sb_2_Te_3_ with a single Dirac cone on the surface. Nat. Phys. 5, 438–442 (2009).

[b17] Lencer D., Salinga M. & Wuttig M. Design rules for phase-change materials in data storage applications. Adv. Mater. 23, 2030–2058 (2011). 21469218 10.1002/adma.201004255

[b18] Chong T. C. *et al.* Phase change random access memory cell with superlattice-like structure. Appl. Phys. Lett. 88, 122114 (2006).

[b19] Chong T. C. *et al.* Crystalline amorphous semiconductor superlattice. Phys. Rev. Lett. 100, 136101 (2008). 18517969 10.1103/PhysRevLett.100.136101

[b20] Chia Tan C. *et al.* Compositionally matched nitrogen-doped Ge_2_Sb_2_Te_5_/Ge_2_Sb_2_Te_5_ superlattice-like structures for phase change random access memory. Appl. Phys. Lett. 103, 133507 (2013).

[b21] Long P., Tong H. & Miao X. Phonon properties and low thermal conductivity of phase change material with superlattice-like structure. Appl. Phys. Expr. 5, 031201 (2012).

[b22] Simpson R. E. *et al.* Interfacial phase-change memory. Nat. Nano. 6, 501–505 (2011). 10.1038/nnano.2011.96 21725305

[b23] Bang D. *et al.* Mirror-symmetric magneto-optical kerr rotation using visible light in [(GeTe)_2_(Sb_2_Te_3_)]_*n*_ topological superlattices. Sci. Rep. 4, 426–432 (2014). 10.1038/srep05727 PMC4101470 25030304

[b24] Tominaga J., Kolobov A. V., Fons P., Nakano T. & Murakami S. Ferroelectric order control of the Dirac–Semimetal phase in *GeTe*-*Sb*_*2*_*Te*_*3*_ superlattices. Adv. Mater. Interfaces 1, 1300027 (2013).

[b25] Yu X. & Robertson J. Modeling of switching mechanism in GeSbTe chalcogenide superlattices. Sci. Rep. 5, 12612 (2015). 26219904 10.1038/srep12612 PMC4518231

[b26] Tominaga J. *et al.* Role of ge switch in phase transition: Approach using atomically controlled GeTe/Sb_2_Te_3_ superlattice. Jpn. J. Appl. Phys. 47, 5763 (2008).

[b27] Tominaga J. *et al.* What is the origin of activation energy in phase-change film? Jpn. J. Appl. Phys. 48, 03A053 (2009).

[b28] Momand J. *et al.* Interface formation of two- and three-dimensionally bonded materials in the case of *GeTe*/*Sb*_*2*_*Te*_*3*_ superlattices. Nanoscale 7, 19136–19143 (2015). 26523888 10.1039/c5nr04530d

[b29] Wang R. *et al.* Toward truly single crystalline GeTe films: The relevance of the substrate surface. J. Phys. Chem. C 118, 29724–29730 (2014).

[b30] Boschker J. E. *et al.* Surface Reconstruction-Induced Coincidence Lattice Formation Between Two-Dimensionally Bonded Materials and a Three-Dimensionally Bonded Substrate. Nano Lett. 14, 3534–3538 (2014). 24810315 10.1021/nl5011492

[b31] Leszczynski M., Szczerbakow A. & Karczewski G. The properties of (Pb, Ge)Te single crystals grown from vapour phase. J. Cryst. Growth 135, 565–570 (1994).

[b32] Johannsen J. C. *et al.* Engineering the topological surface states in the (*Sb*_*2*_)_*m*_ − *Sb*_*2*_*Te*_*3*_ (m = 0−3) superlattice series. Phys. Rev. B 91, 201101 (2015).

[b33] Briois V. *et al.* SAMBA: The 4–40 kev X-ray absorption spectroscopy beamline at SOLEIL. In A. Calisti, C. M. e. S. F. E. (ed.) UVX 2010-10e Colloque sur les Sources Cohérentes et Incohérentes UV, VUV et X ; Applications et Développements Récents, 41–47 (2011).

[b34] Dent A. J. *et al.* Performance of B18, the Core EXAFS bending magnet beamline at Diamond. JPCS 430, 012023 (2013).

[b35] Da Silva J. L. F., Walsh A. & Lee H. Insights into the structure of the stable and metastable (GeTe)_*m*_(Sb_2_Te_3_)_*n*_ compounds. Phys. Rev. B 78, 224111 (2008).

[b36] Ravel B. & Newville M. ATHENA, ARTEMIS, HEPHAESTUS: data analysis for X-ray absorption spectroscopy using IFEFFIT. J. Synchrotron Rad. 12, 537–541 (2005). 10.1107/S0909049505012719 15968136

[b37] IGOR Pro 6.3-Available at: http://www.wavemetrics.com.

[b38] Funke H., Chukalina M. & Scheinost A. C. Wavelet analysis of extended X-ray absorption fine structure data. Phys. Rev. B 71, 094110 (2005).

[b39] Nonaka T., Ohbayashi G., Toriumi Y., Mori Y. & Hashimoto H. Crystal structure of *GeTe* and *Ge*_*2*_*Sb*_2_*Te*_*5*_ meta-stable phase. Thin Solid Films 370, 258–261 (2000).

[b40] Mansour A. N. *et al.* Structural characterization of Bi_2_Te_3_ and Sb_2_Te_3_ as a function of temperature using neutron powder diffraction and extended X-ray absorption fine structure techniques. J. Appl. Phys. 116, 083513 (2014).

[b41] Shportko K. *et al.* Resonant bonding in crystalline phase-change materials. Nat. Mater. 7, 653–658 (2008). 18622406 10.1038/nmat2226

[b42] Pauling L. The Nature of the Chemical Bond (Cornell University Press, Ithaca, NY, 1939).

