# Supplementary material for: Revisiting the Local Structure in Ge-Sb-Te based Chalcogenide Superlattices
Source: Sci Rep. 2016 Mar 1;6:22353. doi: 10.1038/srep22353 (PMC4772802; doi:10.1038/srep22353)
Supplement: Supplementary Information [file srep22353-s1.pdf]

SUPPLEMENTARY INFORMATION

Revisiting the Local Structure in Ge-Sb-Te based Chalcogenide Superlattices

Barbara Casarin[1,2], Antonio Caretta[2], Jamo Momand[3], Bart J. Kooi[3], Marcel A. Verheijen[4], Valeria Bragaglia[6], Raffaella Calarco[6], Marina Chukalina[7], Xiaoming Yu[8], John Robertson[8], Felix Lange[9], Matthias Wuttig[9], Andrea Redaelli[10], Enrico Varesi[10], Fulvio Parmigiani[1,2,11], and Marco Malvestuto[2,*]

[1]Università degli Studi di Trieste, Via A. Valerio 2, 34127 Trieste, Italy
[2]Elettra-Sincrotrone Trieste S.C.p.A. Strada Statale 14 - km 163.5 in AREA Science Park 34149 Basovizza, Trieste, Italy
[3]Zernike Institute for Advanced Materials, University of Groningen, Groningen, 9747 AG, The Netherlands
[4]Department of Applied Physics, Eindhoven University of Technology, P. O. Box 513, 5600 MB Eindhoven, The Netherlands
[6]Paul-Drude-Institut für Festkörperelektronik, Hausvogteiplatz 5-7, 10117 Berlin, Germany
[7]Russian Academy of Sciences, Institute of Microelectronics Technology and High Purity Materials, Moscow, Russia
[8]Engineering Department, Cambridge University, Cambridge CB2 1PZ, UK
[9]Institute of Physics, RWTH Aachen University, 52056 Aachen, Germany
[10]Micron Semiconductor Italia S.r.l., Via C. Olivetti, 2, 20864, Agrate Brianza, MB, Italy
[11]International Faculty, University of Cologne, 50937 Cologne, Germany
[*]marco.malvestuto@elettra.eu

1 Experimental

EXAFS measurements were performed on two X-Ray Absorption Spectroscopy dedicated beamlines, SAMBA and B18, of SOLEIL Synchrotron (FR) and Diamond Light Source (UK), respectively. A high photon flux, with nearly constant intensity within the scanned energy range of $11-11.8$ keV for the Ge K-edge, was provided by a sagittal focusing Si(111) monochromator. By changing the monochromator the available energy range extended to $35-40$ keV, thus covering also the Sb K-edge. The size of the incident beam on the sample was $22x300$ μm^2. Harmonic rejection was achieved by using two collimating/focusing mirrors (Pt- or Ni-coated). In both beamlines, a Ge detector was used to collect data in fluorescence yield (FLY) mode at room temperature, and the angle of incidence was 50°. Six scans ($\sim$ 30 min/scan) per sample were averaged in order to improve the signal-to-noise ratio.[1,2] At SOLEIL, Bragg peaks from the films and the Si substrates were removed from the CSL absorption spectra by continuously rotating the samples with a spinner.

2 Samples Growth

The high-quality $\sim$ 5 mm size single crystal of Sb_2Te_3 was obtained by slowly cooling a molten stoichiometric mixture of high purity 99.999% of Sb and Te via the Bridgman technique in vertical geometry.[3]
The synthesis of high-quality GeTe single crystal of few mm^2 size was performed by melting the pure elements (Ge,Te) in a vacuum-sealed ampoule, where the proportion was Te:Ge=1.002:1. The melt was then cooled down and the ampoule end, in the region of the reaction products, was placed for three days in a furnace (at 600° C). The remaining elementary Te evaporated and condensed in the cold end of the ampoule.[4]
Details about the growth of the CSL sample can be found in the section "Experimental - MBE growth and annealing" of *Momand et al.*[5]

3 Simulations

We computed the simulations of the $\chi(k)$ spectra using FEFF6 code considering the four theoretical models proposed in the literature,[6] whose respective unit cells are sketched in Figure 1 (a) while crystalline parameters are reported in Table 1. All models can be separated into two classes, depending on the character of the separation between the Ge-Te and the Sb-Te layers. The first class presents a full separation of the Ge-Te bilayers moiety from the Sb_2Te_3 quintuple layers (QLs), via weak VdW bonds.[6] The fingerprint of each model is the stacking sequence of the two Ge-Te bilayers. In particular, Ferro-GeTe (F), Petrov (P) and Inverted Petrov (IP) structures have [Ge-Te-Ge-Te], [Ge-Te-Te-Ge] and [Te-Ge-Ge-Te] arrangements, respectively. On the contrary, the second class assumes that mGe-Te units are inserted into one single Sb_2Te_3 QL [Te-Sb-Te-Sb-Te], and that the whole block can be joined via VdW gaps to (n-1) Sb_2Te_3 QLs, like in standard GST alloys.[7] The Kooi (K) model belongs to this class and has m=2 and n=1. Figure 1 (b) displays the $|\chi(R)|_{th}$ simulated spectra for each model compared with the $|\chi(R)|_{exp}$ profiles, at the Ge and Sb K-edges. The calculated spectra $|\chi(R)|_{th}$ spectra, normalized to the main peak, match the experimental $|\chi(R)|_{exp}$ in the first neighbours region but not above 3 Å.

4 White-line Analysis

Figure 2 (a) displays the normalized white lines taken at the Ge and Sb K-edge of the CSL and reference samples. We compared our data with the XANES spectra of GeO, GeO_2 and Sb_2O_3, adapted from *Peng et al.*[8] and *Scheinost et al.*,[9] respectively. It can be noted that - with respect to CSL - each K-edge of the Ge(Sb) oxides is shifted of few eV.
Furthermore, by simulating the FT of the backscattering contribution of the first Ge(Sb) nearest neighbor - i.e. a Te atom, using the KT model - it is possible to observe that this spectrum is composed of two joined peaks at $\sim$ 2 Å and $\sim$2.7 Å (Figure 2 (b)). Therefore, these observations lead to the assumption that in particular the peak at 2 Å in the FFT is not due to oxidation.

5 Data Analysis

EXAFS spectra (Figure 1 insets) were processed and analysed using the IFFEFIT package.[10] Background subtraction from raw absorption data $\mu(E)$, conversion to $\chi(k)$, k-weightening and normalization were performed with ATHENA.[10] The relative Fourier Transform magnitudes (Figure 1 main panels) were obtained using a k-hanning window with Δk= 0.5 Å^{-1} of $2.15-11.2$ Å$^-1$ and $2.35-11$ Å$^-1$ for the Ge and Sb K-edge. In all cases the signal below 1.5 Å was intentionally suppressed by the RBGK card (see ATHENA manual) since a bond length of atomic pairs could not be smaller than this value. The EXAFS data back Fourier Transforms $\chi(q)$ were extracted applying an R-Hanning window between $2-4.5$ Å and then were fitted with ARTEMIS.[10] The procedure employed a linear combination of theoretical backscattering paths generated with FEFF6,[10] making use of 7 Å clusters of the following structural models: (i) for GeTe - Space group R3m, $a = 4.281$ Å,[11] (ii) for Sb_2Te_3 - Space group R-3m, $a = 4.25$Å;[12] for CSL - KT model.[5] Single scattering paths with R_{eff} up to 4.5 Å and with a theoretical scattering amplitude higher than the 10% with respect to the first path, were included. The fitting procedures on the single crystals provided results in good agreement with literature.[13–16] We carried out a multiple K-edges simultaneous analysis on the CSL data to decrease the number of degrees of freedom. In particular, for a given Ge-Sb bond its theoretical radial distance R_{eff} and its relative Debye-Waller factor σ^2 were assumed to vary alike, regardless of which was the absorber. For each edge, we considered one amplitude reduction term S_0^2 and one inner potential correction ΔE_0. The number of neighbours N was constrained by the degeneracy values based on the symmetry of the KT model structure. In addition, the fit was performed in parallel with three different k-weights (1, 2 and 3), in order to tackle both contributions at low and high k-values. Therefore, the EXAFS results could be obtained more reliably not only for the first nearest neighbour pairs, but also for the second nearest neighbours pairs. Best fit values of structural parameters obtained by EXAFS analysis are reported together with their uncertainties in Table 2S, 3S and 4S.

6 Wavelet Transform Analysis

The Wavelet Transform (WT) is a mathematical complete transformation of a signal. Regarding a k^3-weighted EXAFS data, the expression of the WT is given by:

$$W_f^{\psi}(r,k) = \sqrt{2r}\int_{-\infty}^{+\infty} \chi(k')k'^3\psi^*[2r(k'-k)]dk'$$

where r corresponds to the abscissa of the FT of the EXAFS signal ($\chi(k')$) and $\psi^*[2r(k'-k)]$ is the wavelet mother function. In particular, ψ is the Morlet function, which is a complex sine wave confined in a Gaussian envelope:

$$\psi(t) = \frac{1}{\sqrt{2\pi\sigma}}(\exp i\eta t - \exp -\eta^2\sigma^2/2)\exp -t^2/2\sigma^2$$

The parameter η is the frequency of the sine or cosine function, defining how many oscillations there are in a Gaussian envelope with FWHM of σ.[17] All computations were accomplished with the IGOR program for the WT analysis, written by Dr. M. Chukalina and Dr. H. Funke, which can be downloaded at <www.esrf.fr/exp_facilities/BM20/Software/Wavelets.html>. We used $\eta = 30$ and $\sigma = 0.25$, in order to obtain a very high resolution in k-space rather than in R-space.

References

1. Briois, V. *et al.* SAMBA: The 4–40 kev X-ray absorption spectroscopy beamline at SOLEIL. In A. Calisti, C. M. e. S. F. E. (ed.) *UVX 2010 - 10e Colloque sur les Sources Cohérentes et Incohérentes UV, VUV et X ; Applications et Développements Récents*, 41–47 (2011).
2. Dent, A. J. *et al.* Performance of B18, the Core EXAFS bending magnet beamline at Diamond. *JPCS* **430**, 012023 (2013).
3. Johannsen, J. C. *et al.* Engineering the topological surface states in the $(Sb_2)_m$–Sb_2Te_3 (m=0–3) superlattice series. *Phys. Rev. B* **91**, 201101 (2015).
4. Leszczynski, M., Szczerbakow, A. & Karczewski, G. The properties of (Pb, Ge)Te single crystals grown from vapour phase. *J. Cryst. Growth* **135**, 565 – 570 (1994).
5. Momand, J. *et al.* Interface formation of two- and three-dimensionally bonded materials in the case of $GeTe/Sb_2Te_3$ superlattices. *Nanoscale* **7**, 19136–19143 (2015).
6. Tominaga, J., Kolobov, A. V., Fons, P., Nakano, T. & Murakami, S. Ferroelectric order control of the Dirac−Semimetal phase in $GeTe$ - Sb_2Te_3 superlattices. *Adv. Mater. Interfaces* **1**, 1300027 (2013).
7. Da Silva, J. L. F., Walsh, A. & Lee, H. Insights into the structure of the stable and metastable $(\mathrm{GeTe})_m(\mathrm{Sb_2Te_3})_n$ compounds. *Phys. Rev. B* **78**, 224111 (2008).
8. Peng, M. *et al.* Electronic structure and photoluminescence origin of single-crystalline Germanium Oxide nanowires with green light emission. *J. Phys. Chem. C* **115**, 11420–11426 (2011).
9. Scheinost, A. C. *et al.* Quantitative antimony speciation in shooting-range soils by EXAFS spectroscopy. *Geochim. Cosmochim. Acta* **70**, 3299 – 3312 (2006).
10. Ravel, B. & Newville, M. ATHENA, ARTEMIS, HEPHAESTUS: data analysis for X-ray absorption spectroscopy using IFEFFIT. *J. Synchrotron Rad.* **12**, 537–541 (2005).
11. Nonaka, T., Ohbayashi, G., Toriumi, Y., Mori, Y. & Hashimoto, H. Crystal structure of $GeTe$ and $Ge_2Sb_2Te_5$ meta-stable phase. *Thin Solid Films* **370**, 258–261 (2000).
12. Anderson, T. L. & Krause, H. B. Refinement of the $\mathrm{Sb_2Te_3}$ and $\mathrm{Sb_2Te_2Se}$ structures and their relationship to nonstoichiometric $\mathrm{Sb_2Te_{3-y}Se_y}$ compounds. *Acta Cryst. B* **30**, 1307–1310 (1974).
13. Bang, D. *et al.* Mirror-symmetric magneto-optical kerr rotation using visible light in $[(\mathrm{GeTe})_2(\mathrm{Sb_2Te_3})_1]_n$ topological superlattices. *Sci. Rep.* **4**, 426–432 (2014).
14. Mansour, A. N. *et al.* Structural characterization of $\mathrm{Bi_2Te_3}$ and $\mathrm{Sb_2Te_3}$ as a function of temperature using neutron powder diffraction and extended X-ray absorption fine structure techniques. *J. Appl. Phys.* **116**, 083513 (2014).
15. Fons, P. *et al.* The phase-transition in GeTe revisited: Local Versus Average Structure. In *European\Phase Change and Ovonics Symposium - E\PCOS* (2010).
16. Tani, K., Yiwata, N., Harigaya, M., Emura, S. & Nakata, Y. EXAFS study of Sb–Te alloy films. *J. Synchrotron Rad.* **8**, 749–751 (2001).
17. Funke, H., Chukalina, M. & Scheinost, A. C. Wavelet analysis of extended X-ray absorption fine structure data. *Phys. Rev. B* **71**, 094110 (2005).

	a (Å)	c (Å)	χ^2
Ferro-GeTe (F)	4.23	19.14	19.61
Petrov (P)	4.27	17.70	12.63
Inverted Petrov (IP)	4.23	18.71	13.55
Kooi (K)	4.12	17.20	14.52
Kooi TEM (KT)	4.25	41.26	12.34

Table 1. Crystalline parameters a and c of: (i) the theoretical models as in *Tominaga et al.*;[6] (ii) the experimental TEM averaged structure as in *Momand et al.*.[5] χ^2 values from a comparison between theoretical models[6] and experimental CSL data.

**GeTe**	N	R(Å)	σ^2 (Å^2)
Ge-Te$_{short}$	3	2.80(1)	0.007(1)
Ge-Te$_{long}$	3	3.14(1)	0.013(1)
Ge-Ge$_{out-p}$	6	4.12(1)	0.016(3)
Ge-Ge$_{in-p}$	6	4.30(1)	0.016(4)
$R_{factor} = 0.0032$	$S_0^2 = 0.45(2)$	$\Delta E_0 = 4.8(2)$	

Table 2. Local structure parameters for single crystal GeTe. Uncertainties are included in parentheses.

**Sb_2Te_3**	N	R(Å)	σ^2 (Å^2)
Sb-Te$_{short}$	3	2.95(2)	0.009(2)
Sb-Te$_{long}$	3	3.10(3)	0.015(7)
Sb-Sb$_{in-p}$	6	4.20(3)	0.014(6)
Sb-Sb$_{out-p}$	6	4.32(6)	0.012(8)
$R_{factor} = 0.0062$	$S_0^2 = 0.65(7)$	$\Delta E_0 = 5.5(4)$	

Table 3. Local structure parameters for single crystal Sb_2Te_3. Uncertainties are included in parentheses.

**CSL**		N	R(Å)	σ^2 (Å^2)
Ge-Te$_{short}$	B	3	2.81(1)	0.011(1)
	C	3	2.93(1)	0.011(1)
	A	6	2.81(1)	0.011(1)
Ge-Te$_{long}$	B	3	3.09(2)	0.012(2)
	C	3	3.15(2)	0.012(2)
Sb-Te$_{short}$	C	3	2.93(2)	0.008(1)
	A	3	2.95(1)	0.008(1)
Sb-Te$_{long}$	C	3	3.15(3)	0.014(1)
	A	3	3.13(3)	0.014(1)
Ge-Ge$_{out-p,short}$			3.94(3)	0.011(5)
X-X$_{in-p}$	Ge-Ge		4.17(3)	0.009(4)
	Ge-Sb		4.17(3)	0.009(4)
	Sb-Sb		4.17(3)	0.014(8)
X-X$_{out-p,long}$	Ge-Ge		4.39(4)	0.006(2)
	Ge-Sb		4.39(4)	0.003(1)
	Sb-Sb		4.39(4)	0.013(5)
R$_{factor}$(Ge)= 0.0036	S_0^2(Ge)= 0.78(4)		ΔE_0(Ge)= 4.5(2)	
R$_{factor}$(Sb)= 0.011	S_0^2(Sb)= 0.8(1)		ΔE_0(Sb)= 7.9(8)	

Table 4. Local structure parameters for CSL. Uncertainties are included in parentheses.

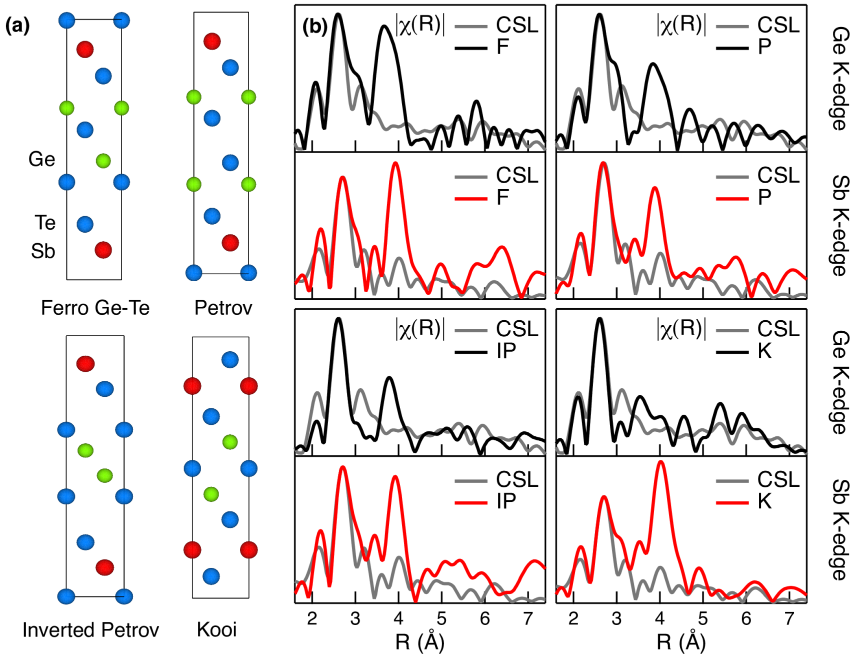

Figure 1. (a) Literature theoretical structures from *Tominaga et al.*[6] and therein. Sb, Te and Ge atoms are denoted with red, blue and green circles, respectively. (b) $|\chi(R)|_{exp}$ of the experimental EXAFS data at Ge and Sb K-edge (grey curves) in comparison with the *ab-initio* simulated $|\chi(R)|_{th}$ (Ge-black, Sb-red curves) of the four models shown in (a).

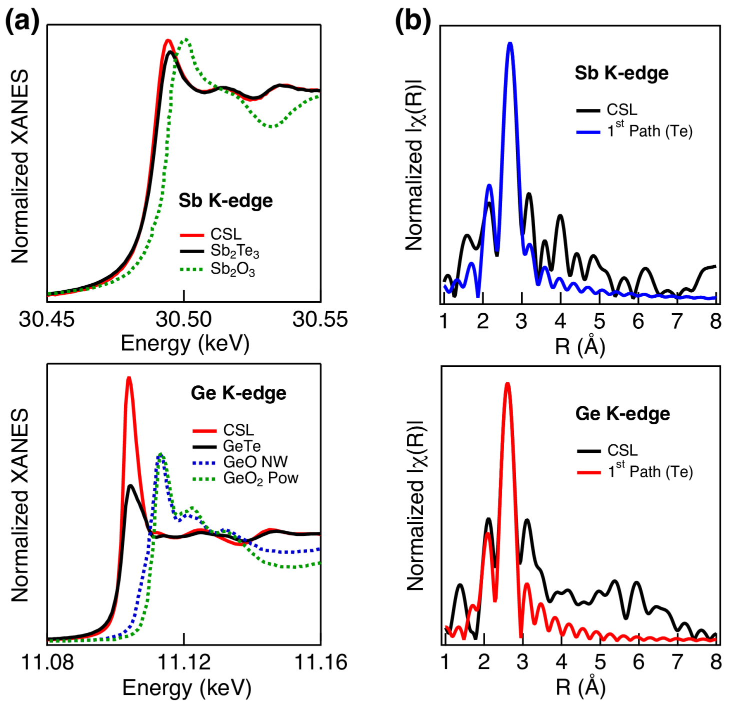

Figure 2. (a) Comparison between the $|\chi(R)|$ of CSL and the simulation of first backscattering path (Te atom) using the KT model, at both Ge and Sb K-edges. The joined-double-peak feature is visible at 2 and 2.7 Å. (b) Comparison between the white lines (i.e. XANES) at Ge and Sb K-edge of CSL and GeO (Nano Wires), GeO_2 (Powder) [Adapted from *Peng et al.*,[8] with permission from Copyright 2011 American Chemical Society] and Sb_2O_3 [Adapted from *Scheinost et al.*,[9] with permission from Copyright 2006 Elsevier]. No oxidation is present in CSL and reference samples.
